# Information leaflets vs artificial intelligence: comparing perceptions of stroke survivors and professionals in a mixed-methods study

**DOI:** 10.1093/esj/aakag037

**Published:** 2026-04-23

**Authors:** Lucie Tvrda, Jennifer K Burton, Katie McConnell, Kalliopi Mavromati, Hendrik Knoche, Robert Mikulik, Terence J Quinn

**Affiliations:** School of Cardiovascular and Metabolic Health, University of Glasgow, Glasgow, United Kingdom; School of Cardiovascular and Metabolic Health, University of Glasgow, Glasgow, United Kingdom; School of Cardiovascular and Metabolic Health, University of Glasgow, Glasgow, United Kingdom; School of Cardiovascular and Metabolic Health, University of Glasgow, Glasgow, United Kingdom; Department of Architecture, Design and Media Technology, Aalborg University, Aalborg, Denmark; International Clinical Research Centre, St. Anne’s University Hospital in Brno and Health Management Institute, Brno, Czech Republic; School of Cardiovascular and Metabolic Health, University of Glasgow, Glasgow, United Kingdom

**Keywords:** artificial intelligence, health education, stroke

## Abstract

**Introduction:**

Stroke survivors describe insufficient access to information post-discharge. We aimed to compare user perceptions of stroke information from third-sector stroke websites with those generated by a widely accessible artificial intelligence (AI) tool and to summarise the attributes of the preferred stroke information formats.

**Patients and methods:**

UK third-sector stroke websites were searched for materials relevant to 15 questions asked by stroke survivors. ChatGPT-4o was used to generate responses. Stroke professionals (clinicians, researchers), stroke survivors and caregivers reviewed third-sector and AI responses, guessing the source and identifying their preferred text. Participants rated responses on scales of empathy, trustworthiness, reliability, comprehensibility and usefulness and provided justification. Proportions of preference and correct guesses, as well as mean ratings, were compared between groups. Framework analysis was used to identify the attributes of response formats preferred by stroke survivors.

**Results:**

Relevant responses were found for 13 (87%) of 15 questions. Across groups, 60 participants with a mean age of 44 (SD = 14) and 57% females, correctly identified 184/300 (61%) of AI responses, and preferred AI in 123/300 (46%) of the cases. The groups differed in preference, with clinicians being least likely to choose AI (34%), followed by stroke survivors (49%) and researchers (54%). Stroke professionals viewed third-sector responses as more empathetic. The themes of content, structure and tone of responses were described with an emphasis on clarity, conciseness and an approachable tone.

**Discussion:**

Artificial intelligence-generated responses to stroke questions were rated positively by stroke survivors and researchers, whereas stroke clinicians were more sceptical.

**Conclusion:**

Widely accessible generic AI tools have the potential to complement existing stroke information resources.

## Graphical Abstract

**Graphical Abstract:**
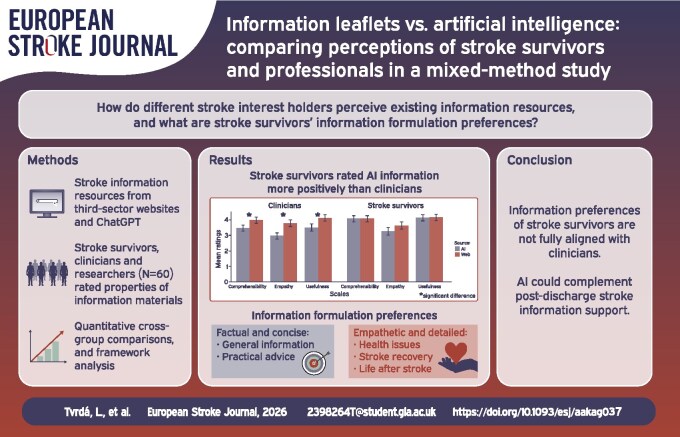

## Introduction

Timely and reliable information is essential to support decision-making after stroke and to improve long-term outcomes.^1^ Stroke survivors and their families frequently report limited access to information following hospital discharge.^2^ Information delivered by healthcare professionals is preferred; however, insufficient communication may lead to unmet information needs.^3^

Third-sector services offer stroke information leaflets created by healthcare and other professionals. The American Medical Association (AMA) recommends that healthcare materials are produced at a sixth-grade reading level to increase the accessibility of health education.^4^ While a helpful resource, current stroke information leaflets often exceed this level, rendering the information difficult to understand.^5^ This impacts stroke survivors’ health literacy, which may lead to poorer health outcomes.^5^

Large language models (LLMs) are increasingly used in healthcare due to their ability to mimic natural language.^6^ An LLM flagship, ChatGPT, has performed well in producing clear and concise medical summaries,^7^ and ensuring comprehensibility by adjusting the text to the literacy level of a reader with a medical condition.^8^ Being widely accessible, ChatGPT can be used by the general public, including stroke survivors and their families. However, clinicians have criticised the accuracy of artificial intelligence (AI)-generated medical information.^9^ General-purpose LLMs may not be trained on trusted medical data and can provide unreliable information while appearing confident.^10^ This, in turn, may harm the level of human trust and pose risks if used to inform medical decisions.^11^

Studies have found that the general population perceives AI-generated medical advice as less empathetic and reliable than that written by a human.^12^ Conversely, people living with a medical condition thought generic AI provided more empathetic responses than healthcare professionals.^13^ Stroke survivors have reported perceived benefits of AI and digital tools in stroke care (eg, digital home rehabilitation),^14^ but their perceptions of current publicly available AI tools as a source of stroke information remain unknown.

This study aimed:

(1) To identify the most common questions following stroke and evaluate the availability of responses found on UK online third-sector stroke websites.
(2) To compare the perceptions of stroke survivors, researchers and stroke clinicians rating stroke information materials produced by current publicly available resources (third-sector stroke websites and ChatGPT-4o).
(3) To summarise the formulations of stroke information across common topics in a way that meets the needs of people with lived experience of stroke.

## Patients and methods

### Ethics and recruitment

This mixed-methods study followed the Strengthening the Reporting of Observational Studies in Epidemiology (STROBE) and the Standards for Reporting Qualitative Research (SRQR) guidelines (Supplementary) and was approved by the University of Glasgow College of Medical, Veterinary and Life Sciences Ethics Committee (200220163).

Participants were recruited by convenience sampling via the NHS Research Scotland Stroke User Group of stroke survivors and professionals.

We included adults who self-identified as: (1) a stroke survivor, a carer or a family member of a stroke survivor; (2) a stroke researcher or (3) a stroke healthcare professional. We aimed for inclusive recruitment with no exclusions around stroke-related impairments. Participants with no access to digital technology were offered in-person interviews.

### Materials and procedure

We compiled a list of commonly asked stroke questions, drawing together interviews with stroke survivors and their families,^15^ including the testing of a prototype chatbot device, and the UK Stroke Association helpline log listing the most frequent enquiries between the years 2023 and 2024 (Table S1). From the aggregated data, we identified 15 questions, ie, the 3 most common questions across 5 overarching topics: general information, health issues, stroke recovery, life after stroke, support after stroke.^3^

We mapped the availability of responses to each of our 15 questions on 4 UK third-sector stroke websites (Stroke Association, Northern Ireland Chest Heart & Stroke, Different Strokes, Chest Heart & Stroke Scotland), classified as: not available; not directly available but related information may be found; available. Artificial intelligence had not been used to produce these resources.

Two stroke researchers (L.T. and K.M.C.) compiled responses to the 15 questions from online materials. Where more than 1 website provided relevant information, we selected the one that best covered the question based on agreement within the team. Additionally, we used ChatGPT-4o to generate a response to each of the 15 questions, limiting the length to 300 words with no additional prompts. ChatGPT was chosen due to its superior readability of patient information compared to other LLMs.^16^ Any information that could identify the source was removed.

#### Measures

We collected demographic measures including age, gender and participant group (healthcare professional; researcher; stroke survivor or family member). We assessed attitudes towards AI using the 5-item Likert AI Attitude Scale (AIAS-4).^17^ We further assessed perceptions of responses: reliability, empathy, comprehensibility to someone who had a stroke and willingness to use the information, through 5-point Likert scales ranging from a very negative to a very positive perception (eg, “very unreliable,” “somewhat unreliable,” “neutral,” “somewhat reliable,” “very reliable”).^12^ The fifth scale, relevance, was added following the same format, to evaluate how well the materials answered the question. Healthcare professionals further rated each response on 6-point Likert scales of accuracy and completeness (scored as 1, completely incorrect/incomplete; 2, mostly incorrect/incomplete; 3, partially incorrect/incomplete; 4, partially correct/complete; 5, mostly correct/complete and 6, completely correct/complete).^18^

#### Procedure

Semi-structured interviews were conducted by L.T. between October 2024 and May 2025, in person or online using Microsoft Teams (v. 25241.203.3947.4411.). Interviews were scheduled to last 1 h, with breaks to prevent fatigue. The first 5 interviews were considered pilots to refine the testing session delivery and were included if no major changes were required. Participants rated 2 responses (third-sector website and AI) to 5 randomly selected questions, 1 for each topic, blinded to the source. The 2 responses were presented sequentially in a random order allocated using RStudio (v. 4.1.2).^19^ Enough time was given to the participants to read and rate the response on each of the scales.

Participants guessed the source of the response (third-sector website or AI) and indicated their preference, briefly exploring the reasoning behind their choice. If a participant’s preference matched their guess, this was treated as a “conscious preference” (eg, guessing AI as the source and preferring the AI response). If consented to by the participant, the interviews were audio-recorded and verbatim transcripts were produced. All transcripts were anonymised prior to data extraction.

### Quantitative analysis

Power analysis was conducted using the G^*^Power tool (v. 3.1.9.7.) to identify the minimum sample size to detect a moderate effect at an alpha level of 0.05. Quantitative data were tabulated using Microsoft Excel (v. 2509). Analyses and visualisations were conducted in RStudio (v. 4.1.2).^19^ We used descriptive statistics to assess the distribution of participants’ guesses, preferences and ratings. All missing values were excluded prior to the analysis. A chi-square test was used to compare correct guesses, preferences and conscious preferences across the groups. Paired *t*-tests were used to evaluate the rating differences between groups.

### Qualitative analysis

All transcripts were imported to NVivo (v. 14).^20^ We used the framework analysis approach^21^ with an a priori interest to identify the attributes of responses favoured by stroke survivors and their families.

L.T. initially screened all transcripts to build a foundation for the analytical framework. Any recurrent points and descriptions were noted.

A random sample of 4 transcripts was coded line-by-line by L.T. to identify expressions by which the quality of a stroke response was characterised. These transcripts were also coded by co-investigators (L.T., K.M., J.B.) to capture a wider range of codes and categories (ie, potential response attributes).

After the initial codes were compared, we grouped those into categories forming the analytical framework. This was applied to the remaining transcripts and developed iteratively, gaining its final shape after the last transcript had been coded and indexed.

We constructed a framework matrix for each of the 5 question topics. Each row represented a participant, with a column for each final theme. Individual quotations were imported into the matrices (Tables S2–S6).

Shared patterns of meaning identified from the framework matrices were discussed within the team, and summaries of commonalities and differences of preferred response attributes were described by topic as themes (Table S7).

## Results

### Common stroke questions

Previous interviews with stroke survivors and caregivers (*n* = 38 stroke survivors, 2 caregivers), and Stroke Association helpline log search revealed the 15 most common stroke questions following hospital discharge (Table 1), representing 40% of all the 27,439 helpline enquiries. Relevant online responses were found for 13 (87%) of the questions. There was no dedicated online information resource for 2 questions: “What are the side effects of stroke medications?” and “Is stroke a genetic condition?”.

**Table 1:** Overview of information leaflets available on UK third-sector stroke websites.

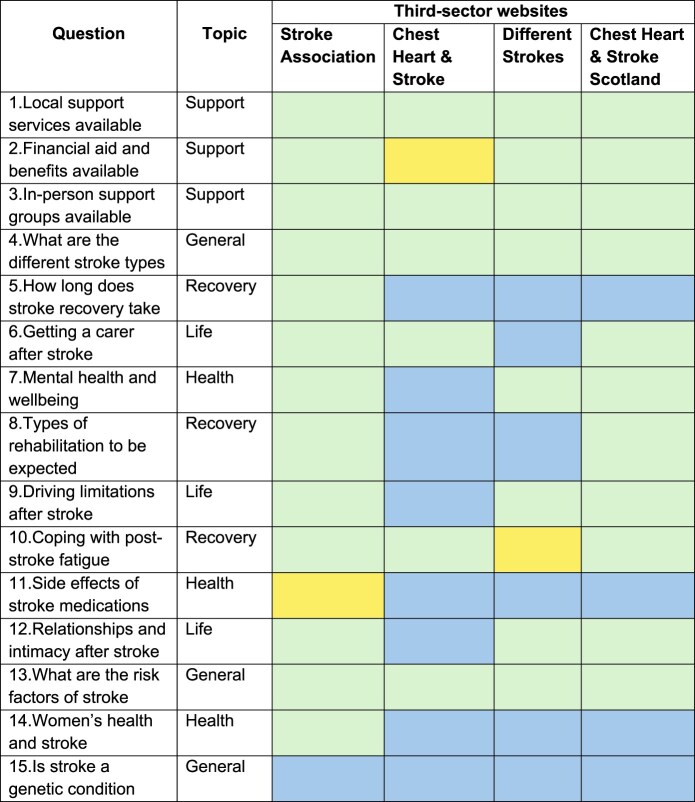

Green = available; blue = not available; yellow = not available but overlap with a different response leaflet. Topics: general information, health issues, stroke recovery, life after stroke, support after stroke.

### Information source preference

We recruited 20 stroke healthcare professionals, 20 stroke researchers, 17 stroke survivors and 3 caregivers/family members. In total, 60 participants (57% female) with a mean age of 44(SD = 14) compared third-sector website and AI responses (Table 2). Four interviews were conducted in-person. The 5 (8.3%) pilot participants did not report their age; otherwise, there were no missing values. Preferences differed significantly across groups (*Χ*^2^(2) = 8.7, *P* < .05) with clinicians being least likely to select AI as their preferred response.

**Table 2:** Descriptive and comparative statistics.

	Clinicians	Researchers	Stroke survivors	Comparative statistic
***n***	20	20	20	
**Age (M, SD)**	43.4 (11.1)	32.3 (9.6)	55.3 (12.1)	
**AI Attitude (M, SD)**	7.66 (1.15)	7.62 (0.91)	5.72 (2.34)	
**Guessed AI correctly (*n*, %)**	66/100 (66%)	65/100 (65%)	53/100 (53%)	*P* = .110
**Preferred AI response (*n*, %)**	34/100 (34%)	54/100 (54%)	49/100 (49%)	*P* = .012
**Consciously preferred AI (*n*, %)**	8/100 (8%)	29/100 (29%)	17/100 (17%)	*P* < .001

*n* = number; M = mean; % = percentage; AI = artificial intelligence; *P* = *P* value; higher values on the AI Attitude Scale represent a more favourable approach towards AI.

**Figure 1:**
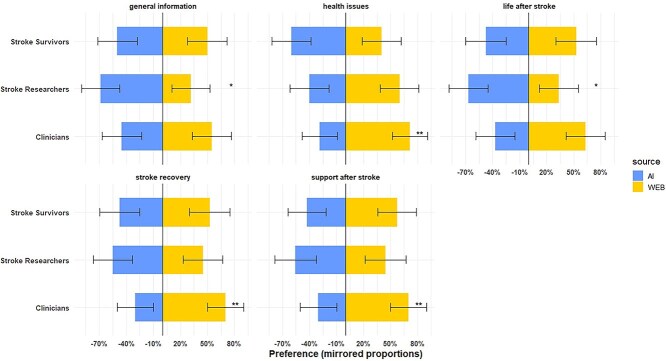
Bar plots representing participant preferences across stroke question topics. % = percent; ^*^ = *P* < .05, ^**^ = *P* < .01; error bars = 95% CI.

When grouped by topic, participant preferences were varied with significantly higher proportions of clinicians preferring third-sector information for the topics of health issues, stroke recovery and support after stroke. AI responses were favoured by researchers for the topics of life after stroke and general stroke information (Figure 1). Stroke survivors did not have a significant preference in any topic.

### Ratings of stroke responses

**Figure 2:**
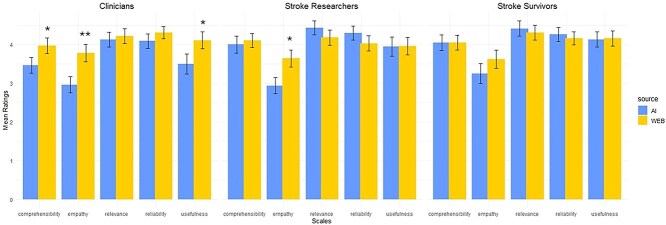
Bar plots showing mean ratings of AI and third-sector WEB materials on 5-point Likert scales of empathy, comprehensibility, usefulness, reliability and relevance. Higher number = more favourable rating. ^*^ = *P* < .05, ^**^ = *P* < .001. Abbreviations: AI = artificial intelligence; WEB = website.

Third-sector responses were rated as significantly more empathetic than AI by clinicians and researchers. Additionally, clinicians were more willing to use third-sector leaflets and rated them as more comprehensible. Stroke survivors’ ratings did not differ between AI and third-sector responses (Figure 2). Out of 100 rating instances, clinicians found 33% of AI responses and 40% of third-sector materials to be completely accurate. Clinicians also rated 32% of AI responses and 23% of third-sector materials as complete. These differences were not significant, as displayed in Table S8.

### Qualitative results

As 2 testing sessions were not recorded, 18 semi-structured interviews with 15 stroke survivors and 3 caregivers/family members were included in the qualitative analysis. Three themes were generated, reflecting preferred response attributes: content, structure and tone (Table 3).

**Table 3:** Framework analysis themes illustrated by quotations.

Quotation no.	Gender, age group (years)	Quotation
**Content**		
**1**	Male, 51–55	*“Response A was really straightforward and down the line and B seemed to sort of meander around a bit more, was a bit more vague. But I definitely like A's presentation more.”*
**2**	Male, 56–60	“*It just kind of said things, said facts, words. It didn’t put any context or meaning to them.”*
**3**	Male, 51–55	“*It was lots of information. You might not need all the information.”*
**4**	Female, 21–25	*“It lists all the different places which I do not know if I did like. So, it’s that I almost did not trust that it was giving specifics anyway.”*
**Structure**		
**5**	Female, 46–50	“*(…) waffly, too much information. It wasn’t relevant(…) and the second one was very clear and concise.”*
**6**	Male, 71–75	“*[…] This is a bit easier to understand, especially for someone like me.(…) my memory is—I’m not as fast, I’m not as reliable reading paragraphs like that”*
**7**	Male, 46–50	“*(…) so it just gives you a list of all the things which I think would be easier for people to understand.”*
**8**	Male, 76–80	“*(…) it was more sort of strict bullet points of boom, boom, boom.”*
**Tone**		
**9**	Female, 56–60	“*Very clinical, not any personable information about it[…]. I’m looking for something, maybe a wee bit of sugar-coated.”*
**10**	Male, 56–60	“*(…) it was more emotional. Mostly, the first one just seemed to give you the steps you would have to follow. The second one seemed to be a wee bit more empathetic.*”
**11**	Female, 71–75	“*I felt it was a bit schoolteacher-ish. You know, you must do this and this because it’s going to make you part of the community […] What if you don’t want a sense of community?”*
**12**	Female, 56–60	“*After having a stroke, the biggest feeling is fear—fear of what’s coming. And then it never said that it would be hard to go to these groups. I found that very hard.*”

#### Theme 1: content

In general, stroke survivors thought that information resources should directly address the question while avoiding irrelevant tangents (quotation 1). Within the individual topics, detailed explanations providing a richer and more meaningful understanding of the information were desired when talking about health issues. For instance, one stroke survivor disliked a third-sector leaflet addressing post-stroke health plainly (quotation 2). Similarly, participants felt that responses to questions about stroke recovery should contain specific actionable advice, information with examples and clear explanations of medical procedures and terms. In contrast, the preferred format of information relating to life after stroke was short and focused (quotation 3). Participants expressed varied needs for information about stroke support. Some preferred a generalised response while others needed a targeted personalised response. However, all agreed that very specific information seemed irrelevant and unreliable (quotation 4).

#### Theme 2: structure

Ease of understanding was perceived as the most desirable quality of the stroke information structure across all question topics. Participants agreed that clear and concise responses with short sentences were preferable (quotation 5), which was also mentioned in the context of post-stroke fatigue and cognitive problems (quotation 6).

The 5 topics differed slightly in the preferred information format, with a bullet-point list being favoured for general information about stroke (quotation 7). Conversely, when talking about stroke recovery, while a clear and concise response was still desired, participants found bullet points overly rigid (quotation 8).

#### Theme 3: tone

Overall, stroke survivors thought that information materials should be written in plain language with an approachable, gentle tone. Excessive use of medical terminology was perceived negatively, with the term “scary” specifically used. Very direct responses were also not favoured (quotation 9).

Within individual question topics, participants preferred warm, empathetic and emotional responses to questions about life and recovery after stroke, as well as health issues (quotation 10). Conversely, an emotional tone in leaflets providing practical information about post-stroke support was perceived as patronising by some (quotation 11). The failure to acknowledge barriers to accessing the support available was also noted (quotation 12).

## Discussion

We have shown that current UK online stroke information materials do not cover all common post-discharge questions, and that stroke interest-holder groups differ in their perceptions of current widely accessible generic AI tools as a source of stroke information. We have highlighted the necessity for materials to be concise, approachable and easy to understand, but also demonstrated differing format and content preferences among stroke survivors, according to the subject matter.

Most healthcare professionals preferred third-sector resources, whereas researchers were more inclined towards AI-based materials, and the preferences of stroke survivors were evenly split. These findings are in line with evidence from other healthcare areas showing that clinicians are more critical of AI-generated content than people living with relevant health conditions, rating it as less comprehensible,^9,22^ and less accurate.^9^

Clinicians and researchers rated third-sector leaflets as significantly more empathetic than generic AI, which is in agreement with previous research where the perception of an empathetic medical response emerged from knowing that the text was human-written.^12^ In our study, participants were blinded to the source of the response, which demonstrates that even if unaware of the authorship, stroke professionals still perceive human-written content as more empathetic. However, an unconscious negative bias towards AI was present, as participants were likely to prefer responses, which they labelled as human-written even if they were, in fact, AI-generated. It should be noted that participants’ acceptability of AI as an information tool may be influenced by the perceived risks relating to personal data retention processes or an unfamiliarity with these policies.

In contrast, stroke survivors’ empathy ratings did not differ between AI and third-sector leaflets. Nevertheless, while expressing a desire for an empathetic tone in health-oriented materials, more than half of stroke survivors favoured AI-generated responses to questions about health issues. In previous research, cancer patients rated responses from a general-purpose AI tool as more empathetic than clinician responses.^13^ This suggests that healthcare professionals’ understanding of empathy may not be fully aligned with the needs of people living with a medical condition.^23^ The importance of empathy in healthcare settings is known, with its reported positive effects on treatment adherence and recovery.^24^ Notably, stroke survivors felt that emotional tone should be used with caution when providing facts and practical information.

Reasoning provided by stroke survivors and their families enabled us to make recommendations for the content, structure and tone of stroke information materials. Specifically, responses to questions about health issues and recovery should be patient-centred, written in a conversational style, contain actionable advice and explain medical facts with empathy. In contrast, general stroke information may be provided as a bullet-point list and remain factual, with a country-specific focus. We recommend that all information materials are clearly structured and concise, using short sentences that are easy to read. We note that this aligns with the characteristics of the sixth-grade reading level recommended by the AMA for all patient-facing healthcare materials.^4^

## Strengths and limitations

The mixed-methods approach allowed us to compare perceptions across groups while gaining a deeper understanding of stroke survivors’ information needs. Moreover, the involvement of stroke researchers sourced perspectives from a group often overlooked in the literature. Due to the hybrid nature of this study, we were able to recruit a wider range of participants, including those with limited access to technology. Our qualitative analysis was purposively focused to make recommendations informed by the participants’ insights.

Despite the inclusive measures, this study was limited by potential selection bias due to convenience sampling, with the participating stroke survivors being younger than the general stroke population,^25^ which may lead to limited generalisability to older, aphasic, cognitively impaired and institutionalised patients. The present sample size is a limitation in that we could not robustly control for confounding factors, such as age, gender and AI attitudes. Furthermore, as we included only frequently asked questions, we did not evaluate the ability of AI to respond to less common enquiries, which are more prone to error.^10^ Participants’ acceptability of AI as an information tool may be maintained due to an unfamiliarity with ChatGPT’s personal data retention policies. It should be noted that, while we strove to utilise the most current AI resources, the version of ChatGPT used in this study had become outdated by the time of submission. Lastly, the present results may not generalise, as only UK websites were included. The United Kingdom is an early adopter of AI, which may have influenced participants’ familiarity with available AI tools and affected the study’s relevance in other European contexts.

## Implications and future directions

The present findings highlight the importance of user involvement in the development of stroke information materials to maximise accessibility and effectiveness. The current recommendations for response formulations may aid with generating prompts to tailor AI responses and with designing novel digital assistive technologies using LLMs to provide targeted stroke information support.

Future studies could further assess perceptions of stroke information formulations using the present recommendations. A larger sample size for the quantitative analysis would enable robust cross-group, and potentially, cross-country comparisons.

## Conclusions

This mixed-methods study has shown that existing online stroke information is incomplete, and the information needs of people affected by stroke are not fully aligned with the opinions of stroke professionals. Artificial intelligence-generated responses were well received by stroke survivors, suggesting the potential for publicly available generic AI tools to complement existing stroke information resources. To increase the accessibility of the information, stroke responses should be clear and concise, with empathy needed to answer health-oriented questions. We emphasise the importance of incorporating the input from people with lived experience of stroke in the development of future information resources.

## Supplementary Material

aakag037_Supplementary_Materials

## Data Availability

Data supporting the present findings are available upon reasonable request.
