# Supplementary material for: Information leaflets vs artificial intelligence: comparing perceptions of stroke survivors and professionals in a mixed-methods study
Source: Eur Stroke J. 2026 Apr 23;11(4):aakag037. doi: 10.1093/esj/aakag037 (PMC13131226; doi:10.1093/esj/aakag037)
Supplement: aakag037_Supplementary_Materials [file aakag037_supplementary_materials.zip › Supplementary Table 8.docx]

**Table 8: Descriptive and comparative statistics of rating scores across groups.**

| **Group** | **Clinicians** | | | **Researchers** | | | **Stroke Survivors** | | |
| --- | --- | --- | --- | --- | --- | --- | --- | --- | --- |
| **Scale** | AI  (M, SD) | WEB  (M, SD) | Comparative statistic (*p*) | AI  (M, SD) | WEB  (M, SD) | Comparative statistic (*p*) | AI  (M, SD) | WEB  (M, SD) | Comparative statistic (*p*) |
| Comprehensibility | 3.46 (1.04) | 3.97 (1.02) | ***p<.*05** | 4 (1.09) | 4.11 (0.92) | *p*=.459 | 4.05 (1.04) | 4.05 (0.98) | *p*=1 |
| Empathy | 2.96 (1.05) | 3.78 (1.13) | ***p<.*001** | 2.94 (1.03) | 3.64 (1.09) | ***p<.*05** | 3.25 (1.32) | 3.62 (1.19) | ***p<.*05** |
| Relevance | 4.13 (0.98) | 4.22 (0.96) | *p=.*500 | 4.43 (0.88) | 4.18 (0.97) | *p*=.162 | 4.41 (1.01) | 4.31 (0.97) | *p*=.281 |
| Reliability | 4.09 (0.94) | 4.31 (0.79) | *p=.*112 | 4.29 (0.89) | 4.03 (0.98) | *p*=.152 | 4.26 (0.92) | 4.16 (0.86) | *p*=.304 |
| Usefulness | 3.50 (1.32) | 4.11 (1.14) | ***p<.*001** | 3.95 (1.22) | 3.96 (1.14) | *p*=.796 | 4.13 (1.00) | 4.16 (1.01) | *p*=.789 |
| Accuracy | 4.93 (1.06) | 5.15 (0.84) | *p*=.059 |  |  |  |  |  |  |
| Completeness | 4.76 (1.2) | 4.73 (1.05) | *p*=.865 |  |  |  |  |  |  |

Note: N=number; M=mean; SD=standard deviation; %=percentage; AI=artificial intelligence; WEB=third sector website; p=p value; higher values on the AI Attitude scale represent a more favourable approach towards AI.
