# Supplementary material for: Information leaflets vs artificial intelligence: comparing perceptions of stroke survivors and professionals in a mixed-methods study
Source: Eur Stroke J. 2026 Apr 23;11(4):aakag037. doi: 10.1093/esj/aakag037 (PMC13131226; doi:10.1093/esj/aakag037)
Supplement: aakag037_Supplementary_Materials [file aakag037_supplementary_materials.zip › Supplementary Table 1.docx]

**Table 1: List of questions commonly asked by stroke survivors after being discharged from hospital.**

| **Question** | **Stroke Association Helpline Log Frequency (N, %)** | **Topic** |
| --- | --- | --- |
| What are the local support services available after stroke? | 1841 (6.7) | Support after stroke |
| What financial aid and benefits is available after stroke? | 1559 (5.7) | Support after stroke |
| What in-person support groups are available in my area? | 1280 (4.7) | Support after stroke |
| What are the different types of strokes? | 939 (3.4) | General information |
| What are the likely stroke recovery outcomes? | 939 (3.4) | Stroke recovery |
| What happens after treatment? How long will recovery take? | 939 (3.4) | Stroke recovery |
| How can I get a carer after stroke? | 915 (3.3) | Life after stroke |
| How does stroke impact mental health? | 746 (2.7) | Health issues |
| What types of rehabilitation can be expected after having a stroke? | 742 (2.7) | Stroke recovery |
| How much rehabilitation will I need? | 742 (2.7) | Stroke recovery |
| What is the timing, intensity, and duration of physiotherapy activities I need to do? | 742 (2.7) | Stroke recovery |
| Will I be able to speak and understand conversation properly again? | 683 (2.5) | Stroke recovery |
| What are the driving limitations after stroke? | 652 (2.4) | Life after stroke |
| How to cope with post-stroke fatigue? | 507 (1.8) | Stroke recovery |
| Why do I feel so overwhelmed and fatigued? | 507 (1.8) | Stroke recovery |
| When can I go back to work after stroke? | 386 (1.4) | Stroke recovery |
| Will I be able to use public transport? | 230 (0.8) | Life after stroke |
| Will I be able to regain my flexibility, balance & endurance? | 223 (0.8) | Stroke recovery |
| Will I be able to coordinate my body movement better? | 223 (0.8) | Stroke recovery |
| Can stroke lead to depression? | 201 (0.7) | Health issues |
| At what point will my movement start to come back in my hand/foot? | 191 (0.7) | Stroke recovery |
| When are blood thinners necessary? How is this decision determined? | 183 (0.7) | Health issues |
| What are the differences between blood thinning medications? | 183 (0.7) | Health issues |
| What are the risks and side effects of blood thinning medications? | 183 (0.7) | Health issues |
| Will I be able to do every day manual tasks (using a knife and fork, opening jars)? | 181 (0.7) | Life after stroke |
| Will I be able to travel and go on holidays? | 127 (0.5) | Life after stroke |
| Will it be hard to get back to running/being aerobically fit again? | 118 (0.4) | Life after stroke |
| What are statins for? | 116 (0.4) | Health issues |
| What combination of lifestyle, medication, and in-hospital treatments/surgery/rehabilitation may be necessary? | 116 (0.4) | Stroke recovery |
| How does stroke affect relationships and intimacy? | 111 (0.4) | Life after stroke |
| Do I need to implement a special diet after stroke? | 105 (0.4) | Life after stroke |
| Why might additional brain imaging be needed? | 96 (0.3) | Stroke recovery |
| Is my blood pressure within the normal range? Can you help me control it? | 81 (0.3) | Health issues |
| Can stroke lead to dementia? | 79 (0.3) | Health issues |
| What are the risk factors of stroke? | 73 (0.3) | General information |
| What are the symptoms of a stroke? | 73 (0.3) | General information |
| What additional tests may I need? | 72 (0.3) | Health issues |
| Do my cholesterol levels put me at risk for a stroke? | 48 (0.17) | Health issues |
| What is atrial fibrillation? | 44 (0.2) | Health issues |
| What are the treatment options for stroke? | 30 (0.1) | Stroke recovery |
| Does diabetes increase my risk of having a stroke? | 21 (0.07) | Health issues |
| How does stroke affect women? | 18 (0.06) | Health issues |
| Is stroke a genetic condition? Did I inherit it? | 15 (0.05) | General information |
| Will my visual problems recede? | 15 (0.05) | Stroke recovery |
| Can you help me quit smoking? | 15 (0.05) | Support after stroke |
| Is smoking associated with stroke? | 15 (0.05) | General information |
| Will I be able to dress myself properly? | 10 (0.04) | Life after stroke |
| What percentage of stroke survivors are independent in daily living? | 7 (0.03) | General information |
| Will I be able to regain a high degree of independence? | 7 (0.03) | Stroke recovery |
| How to know if I will make full recovery? | NA | Stroke recovery |
| Could my past stroke have been prevented? | NA | Health issues |
| Can I prevent having another stroke in future? | NA | Health issues |
| Why did my stroke happen? | NA | Health issues |
| What are the side effects of stroke medications? | NA | Health issues |
| What are the odds that I will have another stroke? | NA | Health issues |
| Is my weight within a healthy range to prevent a stroke? | NA | Health issues |
| Does having a stroke increase my risk of having a heart attack? | NA | Health issues |
| Will the stroke affect how long I have to live? | NA | Health issues |
| Will I be able to wear high heels again? | NA | Life after stroke |
| Can I, or will I, be able to enjoy the quality of life I had before the stroke? | NA | Life after stroke |
| Will I ever return to my pre-stroke situation? | NA | Stroke recovery |
| Will I be able to walk normally again after the stroke? | NA | Stroke recovery |
| Will I be able to write again? | NA | Stroke recovery |
| How much do I need to rely on my own motivation to recover? | NA | Stroke recovery |
| Does the type, severity and site of my stroke impact my recovery potential? | NA | Stroke recovery |
| Will my confidence return? | NA | Stroke recovery |
| Is it true that there is a cut-off point for functional recovery? | NA | Stroke recovery |
| For how long I will receive medication? | NA | Health issues |
| What can you tell me about stroke? | NA | General information |
| What are the causes for ischemic stroke? | NA | General information |
| What are the causes for haemorrhagic stroke? | NA | General information |
| What is a mini-stroke or transient ischemic attack (TIA)? | NA | General information |
| How is a stroke diagnosed? | NA | General information |
| How common are strokes? | NA | General information |

Note: Aggregated data from multiple sources including previous interviews with stroke survivors and their families, and the Stroke Association helpline log of 27,439 enquiries between the years 2023 and 2024. The frequency information is only available for questions documented on the Stroke Association helpline log. N=number; %=percent; NA=not shown on the helpline log.
