# Supplementary material for: Information leaflets vs artificial intelligence: comparing perceptions of stroke survivors and professionals in a mixed-methods study
Source: Eur Stroke J. 2026 Apr 23;11(4):aakag037. doi: 10.1093/esj/aakag037 (PMC13131226; doi:10.1093/esj/aakag037)
Supplement: aakag037_Supplementary_Materials [file aakag037_supplementary_materials.zip › Supplementary Table 2.docx]

**Table 2: Framework matrix showing attributes of responses to questions about general information about stroke.**

| **Participant** | **A : content** | **B : structure** | **C : tone** |
| --- | --- | --- | --- |
| 1: stroke survivor | It was purely factual, and I was asking, the question was asking for facts and I didn't feel it needed to be empathetic in any way. It was just what are the facts? And that's why I preferred it. I felt it was relevant to the question.  Yes, where, where, where there's no ambiguity, I don't think it needs to be empathetic. I think it just needs to be “these are the facts”. What are the risk factors of stroke? And here's your list. And this is it. Yes. So, I didn't feel there was any need for empathy. |  | I was thinking it was scary. |
| 2: stroke survivor |  | I think that one is quite blunt, so it just gives you a list of all the things which I think would be easier for people to understand |  |
| 3: stroke survivor |  |  |  |
| 4: stroke survivor | Didn't seem to contain as much detailed information |  |  |
| 5: carer | I found it easier to understand and I think it gave a little bit more detail. I just when the second one just said, you know TIA is a mini stroke and I just feel like that gives information |  |  |
| 6: stroke survivor |  |  |  |
| 7: stroke survivor | And also it used language like modifiable behaviours and it's kind of like, well, is everyone going to understand what that means?  Yeah, it used very kind of clinical language. |  | It felt quite blunt in the very first kind of paragraph, where it kind of went if you don't get treatment for a stroke, you can die.  Whereas the second one was much better at kind of explaining things in a more meaningful way. |
| 8: stroke survivor | Too much, too much medical information, which was too frightening for people who don't understand |  | Very clinical, not any personable information about it[...]I'm looking for something, maybe a wee bit of sugar-coated.  I found that very scary. |
| 9: stroke survivor | Too many facts, too much information. You don't need all that information. |  |  |
| 10: carer |  |  | just kind of the the language used seems a bit softer. |
| 11: stroke survivor |  |  |  |
| 12: stroke survivor |  |  |  |
| 13: stroke survivor | Lots of sort of medical terminology there, but you need to have the correct terms and I wouldn't fault it |  |  |
| 14: carer |  | It was the second one just read much more easily. It seemed much more on topic. And yeah, it ended up with me having better information, I thought |  |
| 15: stroke survivor |  |  |  |
| 16: stroke survivor | So I'll actually go to take my time and I've got to go back and have a look and maybe highlight and take out the bits that I need and I think. | I think, I think when I’d seen the first layout. And somebody's tried to generate AI answers in there. Then I've had a good look at that and went hold on a minute. This is, that may be relevant or may not be relevant, this is a bit easier to understand, especially someone like me. Not only getting old a bit but someone with uh…like my memories, how should I say it, I'm not, I'm not as fast. I'm not as reliable reading paragraphs like that.  I met the nurse and I spoke to the doctor and I spoke to the stroke people, they were all…brilliant information. Easy to understand which I need. And some, and the way some of the layouts were I found, they seem to go a bit to the side rather than stick with what happened, don’t they? |  |
| 17: stroke survivor |  | The layout was better in the second one. It had a bit of empathy and it was simple to read. Yeah, it was too waffly the first one. |  |
| 18: stroke survivor | Maybe just the fact that it used more kind of medical terms in this one yeah.  It's maybe it's kind of more kind of layman terms |  |  |
