# Supplementary material for: Information leaflets vs artificial intelligence: comparing perceptions of stroke survivors and professionals in a mixed-methods study
Source: Eur Stroke J. 2026 Apr 23;11(4):aakag037. doi: 10.1093/esj/aakag037 (PMC13131226; doi:10.1093/esj/aakag037)
Supplement: aakag037_Supplementary_Materials [file aakag037_supplementary_materials.zip › Supplementary Table 3.docx]

**Table 3: Framework matrix summarising attributes of responses to questions about health issues.**

| **Participant** | **A : content** | **B : structure** | **C : tone** |
| --- | --- | --- | --- |
| 1: stroke survivor | There were things, the thing that struck me was it talked about preeclampsia but didn't explain what it was. And it said at one point about menstrual issues, physical issues with menstruation, and it said go to GP who might recommend an occupational therapist, but it didn't say why. You know, I would have expected to say an occupational therapist who would be able to tell you about specific products or show you alternative methods or…it was sort of, oh, right, but what's the occupational therapist going to do? |  |  |
| 2: stroke survivor | I feel as if the first one had more kind of medical stuff in it because that one (B) was more kind of about different things. |  |  |
| 3: stroke survivor |  | It seemed to be more matter of fact and the way it was, it was laid out |  |
| 4: stroke survivor | It just kind of gave you facts what you need to know, rather than stuff like roundabout more detailed information |  |  |
| 5: carer |  | I feel like it was just very, like structured and laid out whereas this other one was a lot more like longer sentences |  |
| 6: stroke survivor |  |  | It seemed the first one stated facts. Just facts and somebody wrote that they need to be a bit more empathetic. |
| 7: stroke survivor | It kind of looked more formulaic. It it used more technical language. And it just kind of said things, said facts, words. It didn't kind of put any context or meaning on them.  With B it started off with that statement about informal carers, which kind of felt as though it understood the question a little bit better. | And both of them had quite long sentences. But I think with A, I found the sentences just a little bit harder to understand what it was trying to say. |  |
| 8: stroke survivor |  |  |  |
| 9: stroke survivor |  |  |  |
| 10: carer | I felt the second one had more, B had more information, so I think more of a medical professional would have wrote it. |  |  |
| 11: stroke survivor |  |  | the second response just seemed more comprehensive and more empathetic as if it came from a human. |
| 12: stroke survivor |  | It was too, not in depth, but it was more…I don't, I'm not really sure but just A seemed easier to understand and get to the point quicker than B did. |  |
| 13: stroke survivor |  |  |  |
| 14: carer |  |  |  |
| 15: stroke survivor | The only thing which isn't covered in these questions, a lot of it was just referrals on to other people, resources like contact the GP, contact 999, it doesn't, it's not a comprehensive answer. It's more like if you feel like this, do go talk to someone else, but that's not good.  It's not a deep dive. It's sort of an explanation. And then it basically refers you onwards to other information, but it's what it's doing and as well as giving you advice. You know, if you feel suicidal, contacting 999.  I prefer B. There was more information, including percentages in it. |  |  |
| 16: stroke survivor |  |  | It just seemed a little bit more robotic and a little bit more jargony. It didn't seem quite as approachable. |
| 17: stroke survivor |  |  | I know what they're saying is right but I haven't tried some of things, so I suppose I'm very unwilling. Maybe. |
| 18: stroke survivor |  | Well, I'm getting old and that and this information for me that was all very quick. And I don't concentrate the way that I used to concentrate before this happened to me, but I feel that B might have gave me a bit more time to actually see the causes and side effects. |  |
