# Supplementary material for: Information leaflets vs artificial intelligence: comparing perceptions of stroke survivors and professionals in a mixed-methods study
Source: Eur Stroke J. 2026 Apr 23;11(4):aakag037. doi: 10.1093/esj/aakag037 (PMC13131226; doi:10.1093/esj/aakag037)
Supplement: aakag037_Supplementary_Materials [file aakag037_supplementary_materials.zip › Supplementary Table 4.docx]

**Table 4: Framework matrix showing attributes of responses to questions about life after stroke.**

| **Participant** | **A : content** | **B : structure** | **C : tone** |
| --- | --- | --- | --- |
| 1: stroke survivor |  |  | I felt that B was more in layman's terms and also covered…I suppose, I thought it was more empathetic, I think that I would say with B it seemed to be more, it seemed to be less technical and more emotional, personal, yes. I much preferred B. |
| 2: stroke survivor |  |  |  |
| 3: stroke survivor |  |  |  |
| 4: stroke survivor | it was a more kind of generalised rather than specific to an individual person or a patient |  |  |
| 5: carer |  |  |  |
| 6: stroke survivor |  |  | Came across well the second one. It it just, again, it didn't just this is the fact, this the fact. It was more gentle. |
| 7: stroke survivor |  |  |  |
| 8: stroke survivor |  |  |  |
| 9: stroke survivor | It was lots of information. You might not need all the information. |  |  |
| 10: carer | Because it was a bit more information going, giving too much information. |  | It was less precise and a little bit colder. |
| 11: stroke survivor |  |  | it's a language used I felt B had a more empathetic tone to it |
| 12: stroke survivor |  |  |  |
| 13: stroke survivor |  |  |  |
| 14: carer |  |  |  |
| 15: stroke survivor | I'd say I prefer B because that's because it said that you know a care package will be put together by the team at the hospital, whereas A was like you need to contact your local social services department again, which is true, I don't know, but I prefer B I think. |  |  |
| 16: stroke survivor | It just didn't seem to be answering the question. It started off on completely the wrong track. It was answering what will my duties be as a carer for someone, rather than how do I find a carer. |  |  |
| 17: stroke survivor |  |  |  |
| 18: stroke survivor | That seems to be like a one size fits all.  There was a mixture in there that, I don’t know if it was just me, but if I had to read that back I would think that it was more to do with the person's looking after someone. Unless I've not picked that up right, I don't know.  Maybe I'm that, that, there may have been, there may have been information there that's not relevant to me, but again at the same time there may, there should possibly should have been information there that I’ve never seen. | They were quite long, both of them and to me it could have been for the carer or the victim, so to speak.  Yeah, I think I think if there was an AI then it would need to be separated into who you are. |  |
