# Supplementary material for: Information leaflets vs artificial intelligence: comparing perceptions of stroke survivors and professionals in a mixed-methods study
Source: Eur Stroke J. 2026 Apr 23;11(4):aakag037. doi: 10.1093/esj/aakag037 (PMC13131226; doi:10.1093/esj/aakag037)
Supplement: aakag037_Supplementary_Materials [file aakag037_supplementary_materials.zip › Supplementary Table 5.docx]

**Table 5: Framework matrix showing attributes of responses to questions about stroke recovery.**

| **Participant** | **A : content** | **B : structure** | **C : tone** |
| --- | --- | --- | --- |
| 1 : stroke survivor |  |  |  |
| 2 : stroke survivor | I didn't have that type of stroke, so I'm not sure if that's something that should happen or shouldn't, because I didn't he have any any of the stuff that's on that |  |  |
| 3 : stroke survivor |  |  | I think it was probably because it was very, what’s the word, unempathetic. Yeah. It was more sort of strict bullet points of boom, boom, boom. |
| 4 : stroke survivor | I felt B wasn't as kind of personalized, was more of a generic general response, not very specific for certain things. |  |  |
| 5 : carer |  |  |  |
| 6 : stroke survivor |  |  |  |
| 7 : stroke survivor |  |  |  |
| 8 : stroke survivor |  |  | It had a more of a human touch to it, just better understanding of what the issues are. |
| 9 : stroke survivor |  |  | Number one was more factual and not as empathetic as B. |
| 10 : carer | I thought A was more informative. For something so important regarding your driving, more information seem to be more factual and more important to be more factual. |  |  |
| 11 : stroke survivor |  |  | Again, it's just the tone of the language used. I feel like A was more empathetic, B was more clinical. |
| 12 : stroke survivor | I didn't actually prefer one above the other because some of the information in it I didn't agree with, so I don't, didn't really like any of them. |  |  |
| 13 : stroke survivor |  |  | I thought the second one felt more it come from, it was more emotional. Mostly the first one just seemed to give you the instructions as to the steps you would have to follow. The second one seemed to be a wee bit more empathetic. I don't know if that's AI playing a trick or not, but. |
| 14 : carer |  |  |  |
| 15 : stroke survivor | I mean, it's just a description of what people's jobs are. If I literally was typing that into a chatbot and that's what I got. I'll be like, OK, that explained to me the medical profession, not what I'm meant to do now and what I can do.  Again, I take it down a bit because it's acronyms in there. Again, looks more pointed at medical professionals rather than civilians.  It was very relevant, yeah. But again, like not the the information you want after you've had a stroke. So if there was a question, “would this be helpful?”, I don't think it was that helpful.  It looks like it's written by medical medic to sort of like try to explain the profession. And this is difference between an OT and this is a difference between a physio like grand, but that's not really what I want to know.  You'd need something like that if you're being discharged from hospital because your concern would be does my treatment stop? As I said, I'm just like being kicked out. So that's something that would explain that this is part of a sort of a phase and that's useful. | There was that typo type of mistake at the beginning and then there was a sentence that didn't make any sense in the first paragraph that would concern me. | But the way that's written, there's sort of describing things matter of fact, not from a patient point of view. It's too informative, not patient focused. |
| 16 : stroke survivor |  | A was really straightforward and down the line and B seemed to sort of meander around a bit more, was a bit more vague. But I definitely like A's presentation more |  |
| 17 : stroke survivor |  |  |  |
| 18 : stroke survivor | It was the way it was worded. I think I noticed in A, it didn't mention insurance plan, in B it did and I think there was something to do with the are you able to go back and try again about seeing someone? I never noticed that in A, but I'm sure I've seen it in the B. |  |  |
