# Supplementary material for: Information leaflets vs artificial intelligence: comparing perceptions of stroke survivors and professionals in a mixed-methods study
Source: Eur Stroke J. 2026 Apr 23;11(4):aakag037. doi: 10.1093/esj/aakag037 (PMC13131226; doi:10.1093/esj/aakag037)
Supplement: aakag037_Supplementary_Materials [file aakag037_supplementary_materials.zip › Supplementary Table 6.docx]

**Table 6: Framework matrix showing attributes of responses to questions about support after stroke.**

| **Participant** | **A : content** | **B : structure** | **C : tone** |
| --- | --- | --- | --- |
| 1: stroke survivor |  |  | It's a shame you haven't got overly empathetic. It was patronising in places.  It wasn't as patronising as A. Apart from the very first sentence where it says it was crucial to do all these things, and I didn't like that at all. It could be it is advisable. Other words, crucial is you have got to do this and it's just not for everybody at all.  Yeah, some people just, I'm not saying it's right, but some people…I think the information should be out there but tell somebody they've got to do it just eliminates personal choice. And the trouble with having a stroke is your personal choices are restricted anyway.  I felt, I felt it was a bit, again, it was a bit schoolteacher-ish. You know, you must do this and oh, do this because it's going to make you part of the community. And, you know, I mean, somebody like me, the thought of sitting there moaning to everybody else and having to listen to everybody else's problems doesn't appeal.  Yes, I don't like it when you’re told you have to do something. Yeah, fair enough if it's, you know, you're going to die if you don't have this injection. But even then there's an element of choice in that it's just say no, I'm not. Whereas it is crucial that you do this and it is essential. And oh, this wonderful sense of community. What if you don't want a sense of community? |
| 2: stroke survivor | This stuff's relevant, but I'd probably be against like things like YouTube and stuff like that because I did watch a couple of videos myself on YouTube to do exercises and when I spoke to my doctor who's a professional, he said that they don't work. |  |  |
| 3: stroke survivor |  |  |  |
| 4: stroke survivor | It gave an answer for across the board, it seemed to me like it included the answer for other countries, not just where. So it wasn't specifically helping for UK. It was too, too generalized. |  |  |
| 5: carer | It's a bit more like general, whereas like A was very specific. It lists all the different places which I don't know if I did like. So it's I almost didn't trust that it was given specifics anyway. |  |  |
| 6: stroke survivor |  |  | The information is there but it's just laid down in a kind of, this is it, this is it, this is...it doesn't say why. Doesn't seem to be forthcoming and saying "it would maybe help you to do this" or something. It just says go to that association, go to that association. And it's actually off-putting. If I read that, I'd probably go, oh ok, put it to the side. You probably never pick it up again. I think it would depend on the individual but I think after having a stroke, the biggest feeling is fear, fear of what is coming, fear. And then it never said that it can be hard to go to these groups. I found that very hard. |
| 7: stroke survivor |  | You know you can get this service in this town, this town, this town, this town, this town that, which isn't a very helpful way of giving that information and just felt AI generated. |  |
| 8: stroke survivor | A was just kind of like basic facts. B to me appeared to be more of the stuff that you worry about after you've had your stroke and explained a bit in a better way what benefits you can use and where else to go for help. It would give you more information other than what you can get off the Internet.  There was extra stuff in there to help. |  | When you become talking about finance, it's eh, it’s a matter of a fact stuff, isn't it?  B was, had more of a personable touch. |
| 9 : stroke survivor |  | Too much information. And it was a bit confusing. |  |
| 10: carer |  | Yes, number two was waffly, too much information, it wasn't relevant to be sort of a general thing, and the second one was very clear and concise. |  |
| 11: stroke survivor |  |  |  |
| 12: stroke survivor | Well, I think the first one was more geared towards stroke survivors, being part of a peer group and you probably can tell from my answers that I am a member of a peer group. And I think the second one, the AI one, was very general. |  |  |
| 13: stroke survivor | It just didn't seem to hold as much information. |  |  |
| 14: carer | Because more of the information on what you were looking for was given, yeah. |  |  |
| 15: stroke survivor | There was obviously was quite dense information, but that's because the support system is complicated. But it was easy to understand.  If you're in the UK, you want to know about the UK, but you know the thoughts are like it's a compilation of lots of information being pulled together in different countries. |  |  |
| 16: stroke survivor | And just the repeated slightly irrelevant details telling me about services, and Cardiff and Cardigan when I'm not in either of them. It was just a bit, B just felt a bit weird |  |  |
| 17: stroke survivor |  |  |  |
| 18: stroke survivor |  | I just felt B was easier to understand than A. |  |
