# Supplementary material for: Information leaflets vs artificial intelligence: comparing perceptions of stroke survivors and professionals in a mixed-methods study
Source: Eur Stroke J. 2026 Apr 23;11(4):aakag037. doi: 10.1093/esj/aakag037 (PMC13131226; doi:10.1093/esj/aakag037)
Supplement: aakag037_Supplementary_Materials [file aakag037_supplementary_materials.zip › Supplementary Table 7.docx]

**Table 7: Summary of participants' quotations to illustrate key themes.**

|  | **Themes** | | |
| --- | --- | --- | --- |
| **Topic** | **Content** | **Structure** | **Tone** |
| **General information** | Participants favoured detailed but factual and focused information. | Simple, direct list with clear, concise layout is easier to understand. | Excessive use of medical terms felt frightening and difficult to understand. Rephrasing information in plain language is beneficial. |
| **Health issues** | Context and detailed explanation of medical facts should be provided for all recommendations and signposting advice. | Short and clear sentences are preferred and easier to understand. | The tone of the response should be approachable, warm, empathetic and comprehensible instead of plain facts. |
| **Life after stroke** | Clear, specific information without excessive details is desired. The response should stay focused on the question, avoiding irrelevant information. | The response should be short and tailored towards the user category (stroke survivor, carer, family). | The tone of the response should be warm, empathetic and emotional. Plain language was favoured, while avoiding technical terms. |
| **Stroke recovery** | Participants felt that the responses should contain actionable specific advice, information with examples and explanations of medical procedures and terms. | Participants preferred clear, concise and structured layout. In contrast, bullet-point format was not well received. | Participants felt that a warm and empathetic person-centred tone is more relatable to the issues experienced after stroke. |
| **Stroke support** | Participants agreed that very localized information seemed unreliable. Country-specific rather than generalised or very localized responses seem to be optimal. | Participants agreed that the structure of the response should be clear, concise and easy to understand without being overly wordy. | Participants felt that responses may seem patronising and directive. Responses that motivate and suggest options while supporting choice and autonomy are preferred. |
